# Basic Considerations for the Consistency Evaluation Based on ICH E17 Guideline

**DOI:** 10.1007/s43441-024-00737-z

**Published:** 2025-01-06

**Authors:** Meihua Long, Haiyan Wu, Xiaoni Liu, Pengfei Li, Renxin Lin, Ziwei Zhao, Xiujing Kou, Chao Zhu, Chen Ji, Wei Zhang, Kezhou Zhang, Bing Yu, Yun Wang, Hua Zhang, Fan Jia, Yan Hou

**Affiliations:** 1 Department of Biostatistics, Peking University, Beijing, China; 2 Key Laboratory of Carcinogenesis and Translational Research (Ministry of Education), Department of Lymphoma, Peking University Cancer Hospital & Institute, Beijing, China; 3 MSD R&D China, Beijing, China; 4 China Novartis Institutes for BioMedical Research Co., Ltd, Shanghai, China; 5 Takeda Development Center Asia, Shanghai, China; 6 China Innovation Center of Roche, Shanghai, China; 7 Roche Pharma Product Development China, Shanghai, China; 8 Lilly China Drug Development and Medical Affairs Center, Shanghai, China; 9 Sanofi China, Beijing, China; 10 Boehringer Ingelheim China, Shanghai, China; 11 Novo Nordisk China, Beijing, China; 12 R&D-Based Pharmaceutical Association Committee (RDPAC), Beijing, China

**Keywords:** MRCT, Consistency Evaluation, Pooling Strategy, Clinical Trial, ICH E17

## Abstract

In the International Council for Harmonisation (ICH) guidance on General Principles for Planning and Design of Multi-Regional Clinical Trials (E17), it is important to evaluate the consistency of treatment effect across regions in a multi-regional clinical trial (MRCT). In this paper, we elaborated on some basic considerations to evaluate consistency. We first list the design considerations, and then provide consistency evaluation and interpretation on pharmacokinetics, pharmacodynamics, efficacy, safety, and benefit-risk. Furthermore, we consider special situations including non-inferiority, multiple primary endpoints, interim analyses with delayed treatment effect, adaptive design, single-arm studies, rare diseases, and statistical methods for regional treatment effect estimate and consistency evaluation. Finally, if potential inconsistency is anticipated or observed in the MRCT, an exploratory framework is provided for further investigations. Overall, this paper elaborates on consistency evaluation in MRCT, discusses possible challenges in reality and also provides strategies and methods to overcome these challenges. This could help consensus across health authorities, industries, and academic societies, which could further facilitate consistency evaluation and MRCT implementation. Effective communication with regulatory authorities is encouraged to obtain an acceptance of a global approach.

**Supplementary Information:**

The online version contains supplementary material available at 10.1007/s43441-024-00737-z.

## Introduction

ICH E17 [1] was released in November 2017 to provide guidance for MRCT. The purpose of E17 [1] is to outline general principles and strategic issues for planning and designing MRCT. This guideline brings consensus to regulatory authorities across regions/countries so that data from such trials are accepted globally and serve as the primary source of evidence to support the local regulatory approval. Understanding the consistency across different regions helps to inform the regulatory authorities to make decisions.

The ICH E17 outlined analytical approaches to explore consistency evaluation. However, ICH E17 only provides general strategies for handling known factors, unknown factors, and unexpected factors that may cause inconsistency at a high level, but offers limited guidance on the implementation of the guideline. Therefore this paper aims to supplement details in strategic planning and basic considerations to facilitate ICH E17 implementation, building on previous research [2]. First, we describe details for consistency evaluation at different stages of MRCTs, i.e., factors and methods to be investigated over the design, planning, execution, and results interpretation stages. Second, some special considerations are discussed, which were not included in the original guideline. Lastly, we introduce an exploratory framework to investigate the potential inconsistency.

## Study Design Considerations

### Epidemiology, Diagnosis and Current Treatment Landscape

The epidemiological characteristics of the disease are crucial for the overall development of MRCTs, and should be examined carefully for drug development. Epidemiology investigates the disease incidence or prevalence, etiology and risk factors of the disease in the population, histological conditions (e.g., squamous cell carcinoma, adenocarcinoma), mutations (e.g., mutant or wild-type) and prevalence (e.g., EGFR in East Asians), and prognosis. To better understand global epidemiology, summary information on regional differences in overall incidence/prevalence, and stratified incidence/prevalence by various factors are warranted to provide guidance to trial design, such as inclusion and exclusion criteria, stratification factors, and sample size allocation.

Disease background and current treatment landscape may include diagnostic methods, diagnostic criteria, standard of care that may include frontline and late-line therapies, clinical practice, and treatment outcomes. A consistent application of the diagnosis, intended treatment regimen as well as additional or concomitant medication should be specified at the design stage of the MRCT.

### Clinical Pharmacology

MRCT, as an overall strategy for drug development, applies to both exploratory studies in early clinical development and confirmatory studies in the late-stage development. Clinical pharmacology is an important part spanning from exploratory to confirmatory stage studies, including the assessment on pharmacokinetics (PK), pharmacodynamics (PD), and dose-exposure-response (PD, safety, efficacy) relationship, dose optimization and selection, special population and ethnic sensitivity analysis, etc. PK and/or PK-PD obtained in early development studies will assist in identifying regional differences which may impact dose selection. Early phase MRCTs may be considered to understand the impact of ethnicity on PK and/or PK-PD. Based on the ADME (i.e., absorption, distribution, metabolism and elimination) characteristics, genetic data (e.g., genotypes of drug metabolizing enzymes or transporters) in the early studies will help to examine the effects of genetic factors on PK and PD, and recommend if subjects with specific genotypes should be considered a subpopulation. Population PK (popPK) and PopPK-PD approaches and exposure-response (E-R) analysis are usually applied to identify covariates (e.g., race, body weight, age, renal or hepatic impairment, etc.) affecting drug exposure and responses in different populations. Clinical pharmacology assessments provide useful information for dose selection, and thus guide the dose regimens in confirmatory MRCTs in participating population as well as provide a basis for the design, conduct, and interpretation of confirmatory MRCT studies.

In principle, the same dosing regimens should generally be used for all ethnic populations in a confirmatory MRCT. If no important regional differences in PK and/or PD and dose-exposure-response relationships are expected (e.g., drug response may be insensitive to intrinsic and extrinsic factors [3], it is not necessary to obtain these data from subjects in all regions, and the same dosing regimen could be used directly. If significant differences in dose-exposure-response are identified for specific regions/countries or other subpopulations, adjustments in the dosing regimen may be considered under the context of totality of intrinsic and extrinsic factors and dose-response relationships. Caution needs to be taken when using different dosing regimens. An example of leveraging pharmacology in MRCT consistency evaluation design is provided in appendix 1.1.

### Intrinsic and Extrinsic Factors, Pooling Strategy and Sample Size Allocation

Pooling strategies can be used to facilitate consistency evaluation across regions and support regulatory decision-making. In pooling design, there is no uniformly acceptable or optimal approach for sample size allocation in a MRCT. ICH E17 recommends a balanced approach between proportional (in proportion to the size of regions and disease prevalence) and even allocation (allocation of equal number of subjects to each region). One of the big challenges in regional sample size allocation is the selection of consistency evaluation criteria. Considering that consistency evaluation should be conducted in a descriptive framework instead of an official hypothesis testing framework, a holistic approach based on several candidate criteria is recommended over a fixed criterion per ICH E17. Summaries of operational characteristics such as the probabilities of meeting these candidate consistency evaluation criteria could be provided over a range of sample sizes for efficient cross-functional alignment and regulatory communication of sample size decisions. Pooling strategies could provide flexibility for regional sample size allocation and facilitate consistency evaluation.

## Interpretations of the Results

ICH E17 recommends a holistic approach to evaluate regional consistency considering multiple perspectives, instead of relying on results from one criterion or one endpoint. A three-layer method is recommended when interpreting the results.

It is generally recommended to define the pooling strategy in advance. Before the third layer analyses on country/region specific subgroup, it is essential to evaluate consistency across pooled regions and/or subpopulations. It is also important to avoid Simpson’s Paradox when interpreting pooling results [4] using techniques such as standardized method [5].

### Clinical Pharmacology

Key considerations to assess the impact of drug exposure and ethnic factors are listed as follows:

- PK characteristics that include drug exposure (local effects, no or minimal systematic exposure); dose-exposure relationship (linear or complex), metabolic pathways (single or multiple), genetic polymorphisms of relevant drug-metabolizing enzymes or transporters (CYP2C9, CYP2D6, OATP1B1) and drug modalities (e.g., antibodies, RNA).
- PD profiles that include expression or genetic polymorphisms of target (e.g., VKORC, EGFR, HLA), dose-ranging exposure-response relationship profile (flat or steep), pathological mechanism subtype distribution of disease (e.g., HCV), clinical practice (e.g., accessibility of combination, concomitant medications).
- Safety that includes therapeutic window (wide or narrow), risk management measures or monitoring methods and any concomitant medication (such as Chinese herbal medicine).
- Special considerations including rare diseases, pediatric use, etc.

At different stages of clinical development, it is important to utilize model-informed drug research and development. Models (e.g., popPK, popPK/PD, E-R models) developed and validated using local and global study data also help to distinguish the between-subpopulation variability and the between-subject variability to support the consistency evaluation and interpretation of drug dose, usage, efficacy and safety in MRCTs.

### Efficacy/Pharmacodynamics

#### Internal Consistency

Internal consistency refers to consistency of treatment effects across multiple settings (e.g., endpoints, analyses, subgroups) in a MRCT. It can generally be assessed by evaluating the following aspects: the consistency of the primary and sensitivity analyses (across analyses); the consistency of the primary and key secondary endpoints (across endpoints); and the consistency across subgroups. In a MRCT, the pooling strategy is usually adopted to evaluate the consistency of treatment effects across regions or across subpopulations.

#### External Consistency

External consistency refers to whether a consistent treatment effect is observed from multiple data sources. In some cases, multiple trials may be required to support drug submissions and regulatory decisions. When evaluating external consistency, it is common to compare across similar Phase 3 registration trials and/or Phase 2 exploratory trials within and outside of the development program. It is important to investigate whether treatment effects are consistent across trials and to examine the consistent impact of the specific factors, pooling regions, and pooling subpopulations. In addition to presenting each trial separately, integrated efficacy analysis can also be used if needed to describe the overall efficacy profile with all clinical efficacy study data. The pooling strategy may also be considered. The statistical analysis plan for the integrated efficacy analyses should be submitted to the regulatory authorities together with the integrated efficacy analysis report. Sufficient communication with regulatory authorities is encouraged before or during the development of a statistical analysis plan.

### Consistency Evaluation of Safety

When evaluating consistency of safety between the reginal/subpopulation and the overall population, the following key elements should be considered: overall summary of adverse events (AEs), common AEs, severe AEs (≥ Grade 3), serious AEs (SAE), fatal AEs, AEs leading to treatment discontinuation, AEs leading to drug interruption or dose reduction, specific medical events or conditions that are of particular interest in the context of a given drug or therapeutic (as applicable), laboratory data, vital signs, ECGs and other findings. In addition, time to AE onset, duration of AE, outcome of AE, exposure-adjusted incidence rate of AE (as applicable) may be further analyzed as necessary. The main analyses should be determined based on the product safety profile, target population, and disease characteristics etc., and it may not be necessary to repeat every analysis in the overall population for regional/ subpopulation analyses.

The overall strategy includes evaluating the following aspects: (a) whether the safety profile in the study drug group is consistent with the overall population; (b) whether the inter-group differences between the study drug group and the control group in the reginal/subpopulation are consistent with the inter-group differences in the overall population; (c) whether the reported AE categories are similar, etc. Unless pre-specified as study endpoints, post-hoc hypothesis testing comparing safety results between regions is not recommended.

Clinically significant difference is the key to assess whether the safety profile in the regional population is consistent with the overall population. We need to bear in mind that the numerical value in the incidence of AEs between the regional and the overall population does not necessarily indicate a true difference. The reason is that the results may be affected by certain factors, such as the sample size of the overall population and the regional subgroup, etc.

### Consistency Evaluation of Benefit-risk

Based on the previous evaluation, a comprehensive benefit-risk assessment could be conducted for the overall population. Subsequently, the consistency of benefit-risk evaluation between regions and the overall population could be evaluated. The included content is listed as follows:

- Treatment background, e.g., incidence/prevalence, etiology, risk factors, standard of care, prognosis.
- Clinical pharmacology, e.g., PK, PD, exposure-response relationship.
- Benefit, e.g., beneficial endpoints, magnitude of benefits, duration of benefit.
- Risk, such as the nature, incidence, and severity of AEs.
- Benefit-risk assessment.

## Special Considerations

### Non-Inferiority

When superiority trial is not feasible, non-inferiority trial may be considered. Non-inferiority trials should use active control. The goal is to confirm the efficacy of the investigational drug, i.e., the new treatment is not inferior to a clinically unacceptable extent compared to the active control. Non-inferiority trials generally use a fixed margin approach and the non-inferiority margin M needs to be pre-defined in advance in the protocol (use M > 0 for absolute metrics and M > 1 for relative metrics). In such case, the traditional treatment effect may be replaced with non-inferiority margin adjusted treatment effect. Sample size assessment and consistency evaluation can be performed in a non-inferiority study using the same approach as that in a superiority study.

**Table 1:** Non-inferiority margin-adjusted treatment effect

Absolute/Relative indicator	Superior/inferior indicator	Non-inferiority Margin-adjusted Treatment Effect
Absolute Metrics	H_0_: T − C ≤ − M vs. H_1_: T − C > − M (M > 0)	T-C + M
Absolute Metrics	H_0_: T − C ≥ M vs. H_1_: T − C < M (M > 0)	T-C-M
Relative Metrics	H_0_: T/C ≤ 1/M vs. H_1_: T/C > 1/M (M > 1)	T/C*M
Relative Metrics	H_0_: T/C ≥ M vs. H_1_: T/C < M (M > 1)	T/C/M

Absolute metrics include mean difference and rate difference, etc.; relative metrics include rate ratio, hazard ratio, odds ratio, etc

Superior indicators are indicators whose higher values indicate better efficacy; inferior indicators are indicators whose lower values indicate better efficacy

T represents the treatment group effect, C represents the active control group effect, and M represents the non-inferiority margin

### Multiple Primary Endpoints

MRCT may have multiple primary endpoints. There are usually two scenarios: one where treatment effects on all primary endpoints is recommended to establish clinical benefit (co-primary endpoints) and another where treatment effect on at least one of several primary endpoints is sufficient (such as dual primary endpoints). Sample size evaluation should also be adjusted accordingly based on the number of endpoints. For the co-primary endpoints, it is recommended to provide the probability of consistency based on all primary endpoints. For the 2nd scenario such as dual primary endpoints, it is recommended to provide the probability of consistency for each primary endpoint. Consistency evaluation adopts a descriptive framework without hypothesis testing. Multiplicity adjustment for multiple endpoints is not required for consistency evaluation.

### Interim Analyses and Delayed Treatment Effect

MRCT may have one or more efficacy interim analyses which should be pre-specified in protocol. In order to take consistency into account at the design stage, it is ideal to use the same interim analysis plan including stopping rules, alpha allocation and so on. If potential differences exist due to different regulatory agencies, efforts should be made to harmonize the approaches. For consistency evaluation, hypothesis testing at interim analyses and the results of the overall study should all be taken into consideration. It should be noted that the difference in enrollment time in different regions and the difference in sample size for interim analysis may have an impact on consistency evaluation. It is also recommended to provide enrollment status and sample size to facilitate assessment. Follow-up efficacy and safety follow-up data could be provided if needed and feasible. Blinding should be properly handled if there are blinding requirements of subsequent follow-up (primary endpoint not tested yet and/or regulatory requirement). To minimize the impact on the trial and protect trial integrity, there should be a dedicated team responsible for filing, and a separate blinded team responsible for continuing operating the subsequent follow up of the trial.

In some MRCTs, there may be delayed treatment effect due to disease or drug characteristics (e.g., HR = 1 in the first 6 months, followed by HR = 0.7). For example. if there is a delay in regional enrollment, such as 6 months later than global enrollment, it may cause challenges and bias in directly conducting the consistency evaluation. In such case, several supplementary data could be considered such as time-varying piecewise efficacy, longer follow-up data, trial simulations, or data imputation for supportive purpose.

### Adaptive Design

The application of adaptive design in MRCTs has become increasingly popular. Common adaptive designs include sample size re-estimation, group sequential design, removed treatment group designs, and seamless design. The types of adaptations that can be performed include stopping the trial due to futility or efficacy and adjusting for sample size, patient population, treatment group, or patient allocation ratio. Compared with the traditional fixed design, the adaptive design can make the clinical trial more flexible, efficient, and ethical but also more complicated in design, planning, and execution.

During the implementation of an adaptive MRCT, special attention should be taken to ensure that the trial is designed and executed as consistently as possible. In practice, sites in some countries are usually initiated several months later than others due to approval requirements in regulatory, drug supply, and institutional review committees. For the adaptive MRCT, regional sample size allocation should be considered not only for the final analysis but also for interim analyses. If the MRCT is considered to support global simultaneous development, the consideration of the consistency of the final results should not only ensure that it is still applicable under the potential adaptive change of the protocol, but also ensure that it will not change based on the information causing the change of the protocol. Temporal heterogeneity also needs to be considered if it exists. For adaptive MRCTs, computer simulations may be used to determine the operational characteristics to aid trial design and sample size allocation.

### Single Arm Study

Single arm trial (SAT) refers to a clinical trial designed to be open-label without parallel control. Single-arm studies are commonly used in the early exploratory phase of new drugs. In some special cases, SAT may also be considered to support accelerated approval and market launch of new drugs. Applicants are encouraged to communicate this strategy with regulatory authorities to reach alignment using this design. SAT typically uses surrogate endpoints as primary endpoints, such as objective response rate (ORR) in oncology area. Per ICH E17 [1], a holistic approach could be applied to evaluate consistency using multiple candidate evaluation criteria from different perspectives, such as whether the ORR point estimate of region/subpopulation falls within the 95% CI of ORR estimate of the overall population, whether the 95% CI of ORR of region/subpopulation overlaps with that of the overall population, whether the ORR point estimate of region/subpopulation is better than a fixed value, the depth of response, and the duration of response. There is generally small sample size for the overall population and limited enrollment periods in SAT, which could leave some regions/countries very limited enrollment window. In regional sample size allocation, it may be considered to use a pooling strategy to increase sample size flexibly and enhance robustness for consistency evaluation. At the same time, some data borrowing methods, such as Bayesian method, can also be considered to enhance robustness of estimate. It is also recommended to account for the overall consistency evaluation of the clinical pharmacology evaluation.

### Rare Disease

Rare diseases refer to diseases with a very low incidence or prevalence. Drug development for rare diseases is likely to have the following challenges: limited data of epidemiology and the natural course of the diseases, insufficient medical information, possibly lack of established methods and endpoints to assess efficacy, limited clinical study opportunities for patients, limited R&D experiences in this area, multiple subtypes of the diseases, high patient heterogeneity, limited sample size, and narrow enrollment window.

MRCT has historically been used as a quick way to recruit patients with rare diseases and enable global regulatory submissions. Regulatory authorities around the world may have different review criteria for rare diseases. Communication with regulatory authorities should always be performed in advance.

In general, for rare diseases, regional sample size allocation often requires greater flexibility given the challenges. Sample size may be more driven by practical feasibility rather than statistical considerations. Global competitive enrollment, rather than fixed regional sample size allocation, is often used to determine global sample size allocation. Common pooling strategies, such as pooling data from Asia Pacific or East Asia, could be considered. It may be more feasible to use a more flexible approach for consistency evaluation.

### Statistical Models for Analysis

A fixed-effect model (FEM), continuous random-effect model (CREM), and discrete random-effect model (DREM) can be considered for in-depth assessment of regional treatment effects and overall treatment effects as well as variation between regions [6, 7]. When using these models, it is important to evaluate different model assumptions. FEM assumes that both the regional treatment effect and the regional sample size are fixed parameters. CREM assumes that the regional treatment effect is a random variable subject to a certain distribution while the regional sample size is a fixed parameter. DREM assumes that the regional treatment effect is a fixed parameter while the regional sample size is a random variable subject to a polynomial distribution.

**Fig. 1:**
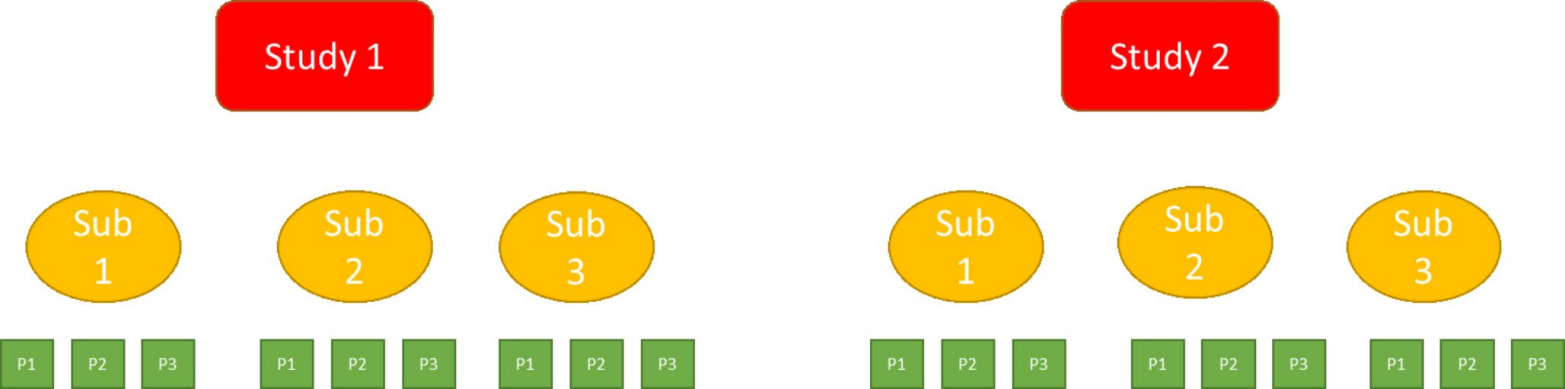
A Hierarchical model structure with three levels: studies, subgroups, and patients

The FEM and CREM are special cases of Bayesian hierarchical models. As shown in Fig. 1, Bayesian hierarchical models are statistical models with multiple levels of detail. Since MRCT contains several regions and patients nested within regions, MRCT are structurally hierarchical and Bayesian hierarchical models can be utilized to estimate treatment effects for each region to reduce variability and improve robustness. For example, FDA’s Center for Drug Evaluation and Research (CDER) statisticians used Bayesian hierarchical model to estimate treatment effects across regions in the Liraglutide Effect and Action in Diabetes [8].

## Exploratory Framework in Case of Potential Inconsistency with Expectations

Even with careful design and appropriate execution, unexpected regional differences may still be observed. If potential clinically relevant differences in treatment effects across regions are observed, a structured and holistic exploration should be utilized to investigate sources of differences. It is also important that consistency evaluation should be conducted using a descriptive framework, rather than a hypothesis testing framework.

### Clinical Relevance

Under the MRCT context, the difference across regions or subgroups may be quantitively large but not clinically relevant considering limited sample size and random highs/lows. Points of consideration include target population, burden of disease, study endpoints, medical practice, sample size, and metrics of treatment effect. If any difference between regions is observed, the first step should be to evaluate whether the regional differences are clinically relevant in terms of size and clinically meaningfulness.

### Disease and Treatment

The summary of epidemics, diagnostics, and treatments in the design and planning stage should be re-examined using the most recent data at the interpretation stage. In some cases, important changes such as trial results update, drug approval, or guideline updates may occur in some regions or countries during the trial. These changes can impact trial execution (withdrawal from trial/treatment, subsequent treatment usage), and subsequently confound the treatment effect.

### Clinical Pharmacology

If clinically significant regional differences in drug exposure, PD and/or dose-exposure-response (PD, safety, efficacy) are identified in clinical studies, in vitro and/or in vivo mechanistic studies should be conducted to explore the impact of PK and PD-related factors, as exemplified in Sect. 2.2. Population PK and PD, M&S and other methods may also be used to analyze the possible impact of each covariate.

### Biological Plausibility

Biological plausibility denotes the degree of causality and relevance of a particular effect on the treatment effect across regions or subgroups that can be predicted or expected. Such anticipation is based on clinical, pharmacological, and mechanistic considerations associated with intrinsic and extrinsic factors. They are also not directly quantifiable or measurable unless they have been accounted at the planning stage. In general, large regional differences are not expected for agents with local effects and targeted therapy for certain genetic mutations. Treatment effects of some drugs may be associated with baseline weight or BMI, baseline risk, histology, and biomarker expression levels, rather than directly with ethnicity or region. This biological plausibility can be used to investigate efficacy and safety, as well as differences between groups, or treatment and control groups.

### Enrollment and Sample Size

In the consistency evaluation exploration, detailed enrollment should be investigated, including overall and regional enrollment start time, enrollment status and end time, regional sample size allocation, proportion of regions/countries of interest and sample sizes of each treatment group. Enrollment affects both exposure time and follow-up time, and the proportion and balance of sample size between groups also affect statistical uncertainty.

### Baseline

Consistency of the baseline of regional population with the overall population between the treatment and the control group should always be evaluated. In addition to pre-defined predictive and prognostic factors, special attentions should be paid to variables where baseline differences and treatment differences were observed. Multivariate models may be considered to adjust for important variables to understand whether differences are caused by baseline imbalances [9]. When the regional sample size is small with many variables, multivariate models may not be applicable. In that case, population resampling method could be considered to assess whether the treatment effect with similar baseline characteristics is consistent between the region and the overall population.

### Exposure, Follow-up, and Disposition

Exposure, follow-up, and disposition including reasons of trial discontinuation and treatment discontinuation should be summarized in detail. For the exposure of combined therapies, it may be considered to include the exposure of each component in the combination therapy. It is also recommended to evaluate the similarity of exposure and study follow-up in the regional and overall population. Differences need to be investigated further about whether there are impacts on the treatment effect and whether exposure or follow-up adjusted analyses should be considered for evaluation.

### Internal Consistency

The strength to support internal consistency is reinforced if internal consistency is demonstrated across with different analytical methods, shown in the different study endpoints (e.g., primary, secondary, and other supportive endpoints); evidenced by generally consistent and stable subgroup results; observed with consistent trends over time. In reality, some observations may appear to indicate potential internal inconsistency but become consistent after further investigation. Details of some examples could be found in appendix 1.2.

### External Consistency

External consistency can be assessed by examining consistency between similar studies, consistency with historical/external data, comprehensive analysis of efficacy, or meta-analysis, as appropriate. Before examining external consistency, it is important to examine whether the trial conditions are similar in terms of treatment regimens, study population, endpoint measures and their summary metrics, as well as intercurrent events and multi-regional context. It is important to identify the potential sources of observed external inconsistency (endpoints, subgroups, analysis methods, treatment differences or certain treatment groups) and adjust properly. Details of some examples could be found in appendix 1.3.

### Statistical Uncertainty

Statistical uncertainty arises from the play of chance. When multiple regions, countries, or subgroups are included in an MRCT, the play of chance can result in observation of seemly inconsistency of treatment estimates (some random highs and lows), particularly when regional sample sizes are small or with too many regions. In such cases, pooling strategies or data-borrowing methods such as Bayesian methods [10] could be considered to reduce variability and increase estimation robustness. The treatment-by-region interaction test could be used to help assessment. In addition, the use of graphics, such as funnel plots, can also be used to display the expected estimate variations (e.g.: 95% CI) under different sample sizes [11]. This helps to better understand regional variability and facilitates further investigation.

### Safety Analysis

When notable differences in safety measures, such as incidence, severity, or category of AEs, are observed between the regional population and the overall population, these differences need to be analyzed further to determine the potential cause. Potential underlying reasons could be caused by PK exposure, intrinsic and extrinsic factors (such as patient weight, baseline, regional medical practice and AE management, concomitant medication like the use of traditional Chinese medicine). If necessary, these analyses could also be combined with previous historical data in regional population. If there are differences in exposure time between regional population and the overall population, exposure-adjusted analyses can be used for safety consistency evaluation, particularly for certain events associated with exposure time.

## Discussions

In MRCT, proper execution is as important as strategy and design. High-quality MRCT requires rigorous and mindful execution, including tracking and adjusting as needed during MRCT. Every effort should be made to start MRCT simultaneously and to reduce the gap between global and local enrollment. Enrollment rate and population characteristics should be monitored timely during the enrollment period. At the same time, special attention should be paid to the consistency of operation across regions in MRCT. It is recommended to use global consistent standard and processes whenever possible, including diagnosis, enrollment, randomization, drug supply, data collection, data cleaning, medical monitoring, quality control, statistical analysis, investigator training, etc. In a word, every effort should be made to ensure consistency in high quality across local trials, to minimize variations in results caused by any hidden factors, to facilitate more efficient drug development, and to increase the chance of submission of marketing authorization to multiple regulatory authorities in different regions simultaneously and to achieve the success of marketing approval.

## Electronic Supplementary Material

Below is the link to the electronic supplementary material.

Supplementary Material 1

## Data Availability

No datasets were generated or analysed during the current study.
