# Supplementary material for: Basic Considerations for the Consistency Evaluation Based on ICH E17 Guideline
Source: Ther Innov Regul Sci. 2025 Jan 6;59(2):328–36. doi: 10.1007/s43441-024-00737-z (PMC11880088; doi:10.1007/s43441-024-00737-z)
Supplement: Supplementary file 1 — Supplementary Material 1 [file 43441_2024_737_MOESM1_ESM.docx]

Appendix

## An example where preclinical and clinical information aid MRCT design involving regional population

In global simultaneous drug development, China’s participation in early MRCT studies may facilitate efficient data collection and improve the assessment across ethnicities and regions. In the case of China subjects participating in MRCT, preclinical PK, PD and toxicology, as well as disease biology, mode of action, clinical operation and local medical practices in the region shall all be considered in designing the study. In addition, the oversea Chinese and Asian data (i.e., PK, PD, efficacy and safety) are also valid evidence for the assessment of differences across ethnicities and regions. In the earlier studies, it is important to comprehensively evaluate the similarity or differences in population between China and the overall regions. If differences are observed, sponsors should consider improving MRCT clinical risk management and control measures or dose adjustment. If necessary, researcher could even conduct clinical pharmacology or other independent trials in the Chinese (or Asian) population to ensure appropriate dose regime for safety and adequate treatment benefit in Chinese subjects.

## Internal Consistency

In practice, the following situations exist. Specific regions are referred to as specific regions/countries here for convenience.

- Results observed for specific regional endpoints differ from those observed for the overall population and this particular regional population differs from the overall population in one important factor. It turns out that in every subpopulation categorized by this factor, the regional population is consistent with the overall population[1].
- Results observed for specific regional endpoints differ from the overall population and this specific regional population differs from the overall population in multiple important factors. Pay special attention to intrinsic and/or extrinsic factors that influence the treatment effect. After covariate-adjusted multivariate model analysis or population resampling method analysis, the regional population is found to be consistent with the overall population if they have similar population profile.
- The primary analyses result for specific regional endpoints differ from those of the overall population. Regional baselines are imbalanced between treatment and control groups. Results became consistent for the regional population and the overall population after applying covariates-adjusted model. Such observed differences may be more attributable to baseline imbalances rather than true clinically relevant differences.
- The results of the primary analysis of specific regional endpoints differ from those of the overall population using the same statistical model. Some complicated models are suitable for large sample size data but not suitable for sample size data. Using the same complicated statistical model on small sample size data may result in non-convergence or non-robustness of the model. Results became consistent after using an optimized model (AIC or BIC or other criterion selection). This observed difference may be caused by the instability of complicated models rather than true clinically relevant differences.
- The results of the analyses of the country-specific primary endpoints differ from those of the overall population. However, results in the pooled region and pooled subpopulation defined by important factors are more robust and were generally consistent with the overall results. In this case, the observed regional differences may be more due to data variability rather than true clinically relevant differences.
- Analyses of the region-specific primary endpoints differ from those of the overall population, but analyses of the important secondary endpoints showed generally consistent and clinically significant improvements from those of the overall population.. This observed difference is more likely due to instability of some metrics (such as subjective metrics) under small regional sample sizes rather than true clinical differences.
- Point estimates for specific regional endpoints differ from those for the overall population, but investigation of trends over time revealed temporal heterogeneity in the pattern of change (e.g., delayed treatment effects). Trends over time for regional populations are consistent with trends over time for the overall populations (e.g., piecewise HR). This observed difference in point estimate summaries is more likely due to differences in follow-up time rather than true clinically relevant differences. Results tend to be consistent under similar follow-up time and tends to be consistent over time.
- Results for specific regional endpoints differ from those for the overall population at a particular time point, but trends over time show that results for the regional population are generally consistent with those for the overall population at both prior and subsequent time points. This observed difference is more likely due to the variability of the data at a specific time point rather than a true clinically relevant difference.
- Point estimates for specific regional endpoints differ from those for the overall population at the interim analysis. However, it is found that there is a trend that as follow-up time increase, the proportion of sample size in specific regions increases, the degree of variation decreases, and the degree of consistency increases. This observed difference may be due to variability caused by the limited sample size at the interim analysis.

## External Consistency

In practice, the following situations exist. Specific regions are referred to as specific regions/countries here for convenience.

- Results observed in specific regional endpoints differ from those for the overall population in certain studies, but not in other similar studies. The integrated analysis of efficacy yields more robust outcomes and the region-specific results are found to be consistent with the overall results.
- Specific endpoint results observed in specific regions differ from those from those seen in the overall population in certain studies. However, the treatment groups are similar and the differences are mainly driven by the control group. However, no specific regional differences or changes that could impact efficacy were observed in historical studies and in contemporary external data. The differences observed in this case may not be true clinical differences.
